# A Case of Dislocation of the Hip-Joint

**Published:** 1857-03

**Authors:** Charles H. Baker

**Affiliations:** Alexander, Genesee Co., N. Y.


ART. VI.—*A Case of Dislocation of the Hip-Joint. Reduced by Reid's Method.* By CHAS. H. BAKER, M. D., Alexander, Genesee Co., N. Y.

Should the report of a case of dislocation of the head of the femur, and its reduction by manipulation, be of use or interest, here you have one:

B. T., a lad of seven years of age, playing on the ice, fell, with a larger boy upon the top of him. He could neither rise unassisted, nor walk when lifted to his feet. I saw him some three hours after the accident. He was lying on his back, with both thighs flexed; the right one could not be moved without producing great pain. The knee was adducted, with the bottom of the foot looking outward. Where the trochanter should have been was flattened, while a prominence on the dorsum ilium indicated its presence where it should not be. I pronounced it a case of dislocation of the head of the femur on the dorsum ilium.

The patient was now placed upon a settee in a recumbent position, and as he was not disposed to be hurt, if he could avoid it, his mother was obliged to hold his hands and press the shoulders down, while the father held firmly the pelvis. I then grasped the knee with my right hand, and the trochanter and pelvis on the right side, with my left; I now carried the knee inward and upward across and near the body. Rotating the limb so as to turn the bottom of the foot inward, and tilt the head of the former forward, I carried the knee down across the left thigh with a slight rotating motion, lifting at the same time, the trochanter with the fingers of the left hand, the thumb resting on the pelvis. I could feel the head of the bone rising around the rim of the acetabulum, when as the right knee was crossing its fellow the head of the femur went, with a snap and jar, into the acetabulum. The limb was now straightened and laid, without difficulty, along with its mate, the patient and myself feeling perfectly relieved and easy. I will only add, the operation was performed in altogether less time than it has taken me to describe it.

